# Supplementary material for: Identification of photoperiod-induced specific miRNAs in the adrenal glands of Sunite sheep (Ovis aries)
Source: Front Vet Sci. 2022 Jul 22;9:888207. doi: 10.3389/fvets.2022.888207 (PMC9354845; doi:10.3389/fvets.2022.888207)
Supplement: Supplementary file 1 [file Data_Sheet_1.ZIP › Supplementary file/miRDeep2 file.zip!/miRDeep2 file/Know_Structure/LP42A1/oar-mir-411a.pdf]

miRBase precursor : oar-mir-411a
Total read count : 32152
oar-miR-411a-5p read count : 30037
oar-miR-411a-3p read count : 2110
remaining reads : 5

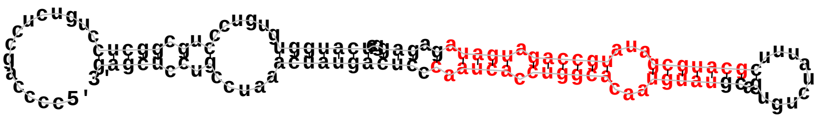

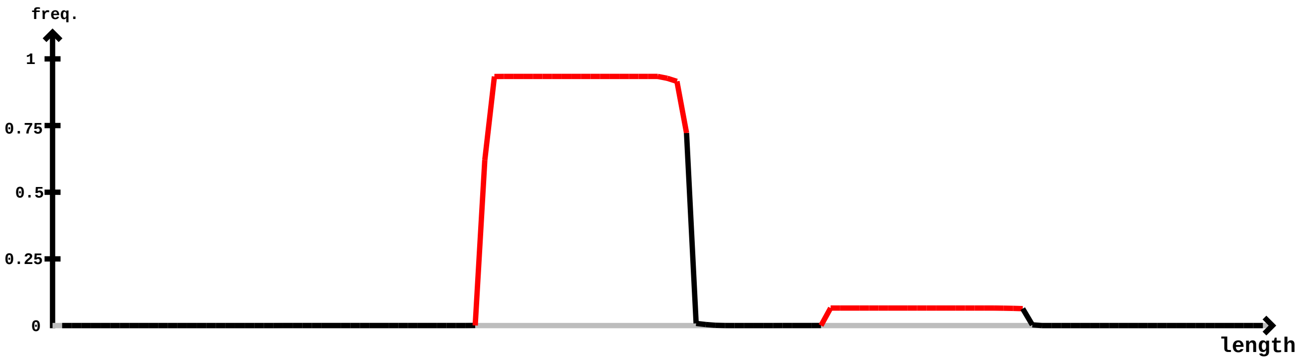

oar-miR-411a-3p
oar-miR-411a-5p

```
5'- ccccagccucuguccucggcgucccugugugguacucggagagauaguagaccguauagcguacgcuuuaucugugacguauguaacacgguccacuaacccucaguaucaaauccguccucgag   -3'    exp
    ..............(((((.(.(......(((((((..(((.(.((((.((((((...((((((((.........).)))))))...)))))).)))).).))))))))))).....).).)))))    reads   mm
    ......................cccugugugguacucggagaga.................................................................................    2       0
    .......................ccugugugguacucggagagau................................................................................    1       0
    ..........................................gauaguagaccguauagcguac.............................................................    2       0
    ..........................................gauaguagaccguauagcguacg............................................................    7       0
    ...........................................auaguagaccguauagc.................................................................    1       0
    ...........................................auaguagaccguauagcg................................................................    1       0
    ...........................................auaguagaccguauagcgu...............................................................    197     0
    ...........................................auaguagaccguauagcgua..............................................................    265     0
    ...........................................auaguagaccguauagcguac.............................................................    4041    0
    ...........................................auaguagaccguauagcguacg............................................................    15341   0
    ...........................................auaguagaccguauagcguacgc...........................................................    72      0
    ...........................................auaguagaccguauagcguacgcu..........................................................    25      0
    ............................................uaguagaccguauagcgu...............................................................    29      0
    ............................................uaguagaccguauagcgua..............................................................    104     0
    ............................................uaguagaccguauagcguac.............................................................    2184    0
    ............................................uaguagaccguauagcguacg............................................................    7597    0
    ............................................uaguagaccguauagcguacgc...........................................................    64      0
    ............................................uaguagaccguauagcguacgcu..........................................................    66      0
    ............................................uaguagaccguauagcguacgcuu.........................................................    27      0
    ............................................uaguagaccguauagcguacgcuuu........................................................    3       0
    ............................................uaguagaccguauagcguacgcuuuaucugugacgu.............................................    1       0
    .............................................aguagaccguauagcguacg............................................................    4       0
    .............................................aguagaccguauagcguacgc...........................................................    1       0
    .............................................aguagaccguauagcguacgcu..........................................................    1       0
    .............................................aguagaccguauagcguacgcuu.........................................................    2       0
    ..............................................guagaccguauagcguacg............................................................    3       0
    ..........................................................................ugacguauguaacacgguccacu............................    1       0
    ..............................................................................guauguaacacgguccacuaac.........................    4       0
    ...............................................................................uauguaacacgguccacu............................    13      0
    ...............................................................................uauguaacacgguccacua...........................    23      0
    ...............................................................................uauguaacacgguccacuaa..........................    36      0
    ...............................................................................uauguaacacgguccacuaac.........................    1945    0
    ...............................................................................uauguaacacgguccacuaacc........................    88      0
    ................................................................................auguaacacgguccacuaac.........................    1       0
```

oar-miR-411a-3p

oar-miR-411a-5p

ccccagccucuguccucggcgucccugugugguacucggagagauaguagaccguauagcguacgcuuuaucugugacguauguaacacgguccacuaacccucaguaucaaauccguccucgag
